# Immuno-PCR - A New Tool for Paleomicrobiology: The Plague Paradigm

**DOI:** 10.1371/journal.pone.0031744

**Published:** 2012-02-09

**Authors:** Nada Malou, Thi-Nguyen-Ny Tran, Claude Nappez, Michel Signoli, Cyrille Le Forestier, Dominique Castex, Michel Drancourt, Didier Raoult

**Affiliations:** 1 Aix-Marseille Université, URMITE, UMR CNRS 6236- IRD 198, Faculté de Médecine, Marseille, France; 2 Aix-Marseille Université, Anthropologie Bioculturelle, UMR 6578 CNRS, EFS, Marseille, France; 3 Institut National de Recherches Archéologiques Préventives UMR 6130, Centre d'Etudes Préhistoire, Antiquité, Moyen Age, Direction Interrégionale Centre, Ile de France, France; 4 De la Préhistoire à l'Actuel: Culture Environnement et Anthropologie - Laboratoire d'Anthropologie des Populations du Passé, UMR 5199, Université de Bordeaux, Bordeaux, France; Charité-University Medicine Berlin, Germany

## Abstract

**Background:**

The cause of past plague pandemics was controversial but several research teams used PCR techniques and dental pulp as the primary material to reveal that they were caused by *Yersinia pestis*. However, the degradation of DNA limits the ability to detect ancient infections.

**Methods:**

We used for the first time immuno-PCR to detect *Yersinia pestis* antigens; it can detect protein concentrations 70 times lower than the standard ELISA. After determining the cut-off value, we tested 34 teeth that were obtained from mass graves of plague, and compared previous PCR results with ELISA and immuno-PCR results.

**Results:**

The immuno-PCR technique was the most sensitive (14 out of 34) followed by the PCR technique (10 out of 34) and ELISA (3 out of 34). The combination of these three methods identified 18 out of 34 (53%) teeth as presumably being from people with the plague.

**Conclusion:**

Immuno-PCR is specific (no false-positive samples were found) and more sensitive than the currently used method to detect antigens of ancient infections in dental pulp. The combination of three methods, ELISA, PCR and immuno-PCR, increased the capacity to identify ancient pathogens in dental pulp.

## Introduction

Since its first description in 1993 with the molecular detection of *Mycobacterium tuberculosis* DNA in an ancient human skeleton [1], paleomicrobiology has become a burgeoning field allowing the identification and characterization of microorganisms (viruses, bacteria and parasites) in ancient specimens [2], [3]. Paleomicrobiology permits the identification of causative agents of past infectious diseases and the temporal and geographical distribution of infected groups and traces the genetic evolution of microorganisms [4]. It benefits modern microbiology by the invention of new diagnostic techniques including the dental pulp study, the suicide PCR and the Multiple Spacer Sequencing Typing (MST) and changes in infectious disease paradigms, including that bovines were not source of prehistorically human tuberculosis [4], [5]. Therefore, paleomicrobiology opens the way for the elucidation of controversies concerning different past infections, such as the plague [6], [7], influenza [8] and tuberculosis [9], [10].

For a long time, the history of the plague was surrounded by many questions concerning the etiologic agent. Based on the description of outbreaks associated with bubonic lesions, 3 devastating plague pandemics have been identified: the Justinian plague (AD541–AD750), the medieval Black Death (which began in Europe in AD1347) and the current pandemic starting in 1855 [4]. Dental pulp is an important source of DNA that has been used in different studies [11]–[14] and showed to be more efficient that bone samples.

In 1998, *Yersinia pestis* DNA was first identified in the dental pulp from 6 of 12 unerupted teeth extracted from skeletons that were excavated from 16^th^ and 18^th^ century French graves resulting from relapses of the medieval pandemic [15]. The first confirmation of *Y. pestis* as the agent responsible for the plague during the early medieval pandemic was obtained in 2000 using suicide PCR, detecting *Y. pestis* DNA in dental pulp of the teeth of children and two adults from the 14^th^ century Black Death pandemic [16]. In addition, *Y. pestis* DNA has been detected in 6 teeth from 2 different skeletons from Aschheim (Upper Bavaria, 6^th^ century), indicating that this bacterium may have been the causative agent of the plague during the Justinian pandemic [17].

Recently, the implication of *Y. pestis* in the Black Death pandemic was confirmed since *Y. pestis Orientalis* DNA has been detected using high throughput multiplexed real-time PCR in 173 dental pulp specimens from Venice dating from the 14^th^ century to the 17^th^ century [18]. Finally in 2010, Haensh *et al* [19] and Schuenemann *et al* [14] confirmed that the circulating genotype of medieval plague was not *medievalis* but more likely a variant related to *orientalis*.

Molecular biology techniques are commonly used to detect microorganismal DNA, but the risk of contamination, chemical modification and fragmentation of DNA and the presence of PCR inhibitors in ancient samples have led researchers to explore alternative methods based on antigenic protein detection. Previous studies have shown that protein sequences can be obtained from the bones of a 160,000–600,000-year-old extinct mastodont and a 68-million-year-old dinosaur, indicating the persistence of proteins across geological time [20], [21]. In addition, the good conservation of proteins has been recently described with the classification of mammalian species using the dental pulp of modern and ancient individuals representing five mammal species including human, from five burial sites from 8,500 year ago using mass spectrometry peptide profiling [22]. *Y. pestis* F1 antigen was detected by 2 different teams using a rapid diagnostic dipstick test (RDT) on putative plague victims exhumed from four archaeological burial sites in southeastern France [23] and from 3 archeological burial sites in Netherland, Germany and Italy [24]. In addition, an Italian team used ELISA and immunohistochemical analysis to identify the F1 antigen of *Y. pestis* in ancient skeletons of plague victims from Venice (San Leonardo in Fossa Mala, 14 ^th^ century) and from Genoa (Bastione dell'Acquasola, 14^th^ century) [25]. However, ELISA is unsuitable for ancient samples due to the detection limit of ELISA and the availability of only small quantities of samples.

**Figure 1:**
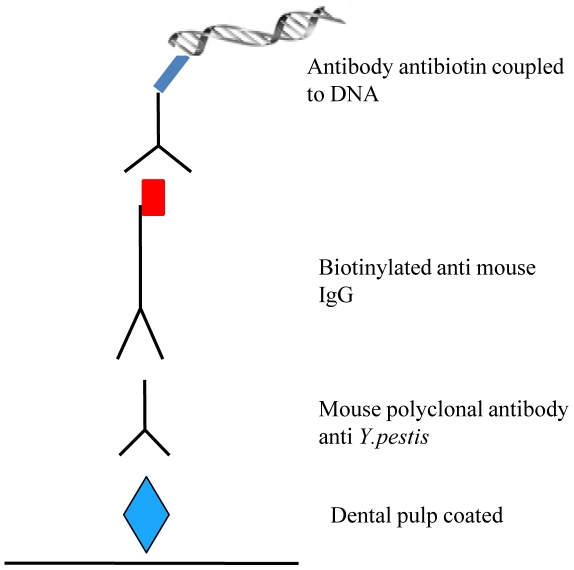
Adaptation of immuno-PCR for the detection of *Y. pestis* antigen in dental pulp extracted from ancient teeth.

To increase the sensitivity of protein detection, we used immuno-PCR for the first time to detect *Y. pestis* proteins. Immuno-PCR (iPCR) was first described in 1992 [26] and is a method that combines the specificity and versatility of ELISA and the amplification power of PCR. Using iPCR, a typical 100- to 10000-fold improvement over the detection limit of the ELISA has been obtained in almost all applications [27], [28]. Since 1993, this method has been applied for the detection of tumor markers, pollutants in the environment, antibodies and viral and bacterial antigens [27]. Technically, the antigens are recognized by a detection antibody that is conjugated to a linker molecule that attaches the antibody-antigen complex to a DNA-tag, which is subsequently amplified by PCR (Figure 1). Here, we present for the first time the adaptation of iPCR for the detection of the plague agent *Y. pestis* in dental pulp specimens collected from Black Death victims to evaluate the potential of iPCR as a method of choice for the detection of pathogenic proteins pathogen in ancient specimens.

## Results

### Determination of the detection limits of ELISA and iPCR for *Yersinia pestis* antigens

**Figure 2:**
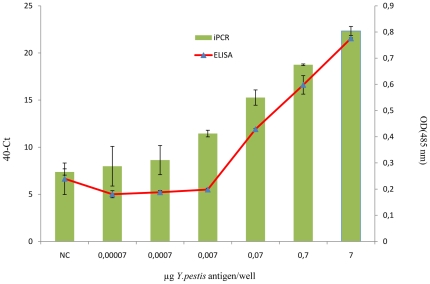
Determination of the detection limits of ELISA and iPCR using various dilutions of the *Y. pestis* antigen. Because the Ct values obtained by iPCR were inversely proportional to the antigen concentrations, delta-Ct values were calculated by subtracting the Ct values from the maximum cycle number of 40. Mean value and standard deviation of 2 different experiments are represented.

The detection limits of ELISA and iPCR were compared using *Y. pestis* that was serially diluted in PBS and directly immobilized in the wells of a microplate (Figure 2). For ELISA, the mean ODs of the *Y. pestis* antigen at concentrations of 70 pg to 7 µg and of the negative control were 0.180±0.01, 0.188±0.007, 0.198±0.004, 0.429±0.002, 0.598±0.03, 0.775±0.002 and 0.239±0.06, respectively. The cut-off value was calculated as the mean of the negative control plus 2SD (standard deviation), resulting in a detection limit of 50 ng of *Y. pestis* antigen diluted in PBS. By iPCR, 7 µg of the *Y. pestis* antigen was detected at 17.66±0.47 Ct, whereas the lowest concentration was detected at 32.01±2.1Ct. The negative control was detected at 32.62±0.33Ct. The cut-off value was calculated as the mean of the negative control minus 2SD resulting in a cut-off value of 31.96 Ct. Using concentrations ranging from 7 µg to 7 ng, the samples were classified as positive for the *Y. pestis* antigen because they exhibited a Ct value below the cut-off value. The detection limit was 0.74 ng using iPCR, representing an enhancement of the detection limit by a factor of 70 compared to the detection limit of 50 ng using ELISA under the same conditions. Once the limits of detection of both methods were determined, ELISA and iPCR were used to detect *Y. pestis* antigens in the dental pulp of ancient teeth collected from the skeletons of individuals with anthropologic and macroscopic evidence of infection.

### Detection of the *Y. pestis* antigen in the dental pulp of ancient specimens

Optimization of ELISA and the iPCR assay and the determination of the cut-off value: An optimization of the different parameters was necessary for the adaptation of ELISA and iPCR for the detection of the *Y. pestis* antigen in ancient specimens. The concentration of dental pulp used to coat the wells was limited by the dental pulp concentration of each sample. After extraction, the concentration of the protein in the dental pulp varied from 0.01 µg/µl to 0.39 µg/µl. We tested coating concentrations from 0.1 µg/well to 0.4 µg/well and chose the coating concentration of 0.4 µg/well. The dilutions for the polyclonal detection antibody anti-*Y. pestis* were based on immunofluorescence results [26], whereas the dilution (1∶1000) for the biotinylated anti-mouse IgG was recommended by the manufacturer for the ELISA experiments.

Determination of the cut-off value: To determine the cut-off value for ELISA, 10 negative teeth, including 5 ancient and 5 modern teeth, were tested. Using ELISA, the OD value varied from 0.102 to 0.181, leading to a mean value of 0.140±0.027 and a cut-off value of 0.196. Using iPCR, the 10 negative teeth yielded a Ct value that varied from 24.12 to 27.89, leading to a mean Ct value of 26.71±1.42 and a cut-off value of 23.86 Ct (ΔCt = 16.13).

**Figure 3:**
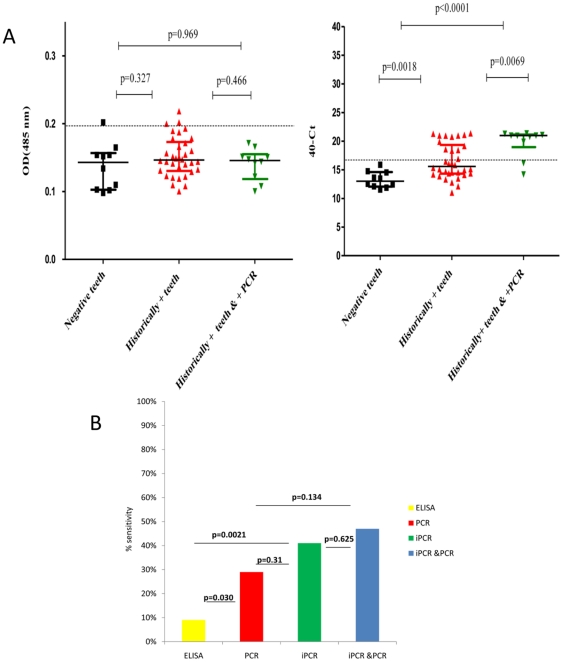
A: Blind detection of the *Y. pestis* antigen in 46 coded teeth, including 34 historically positive teeth and 10 negative teeth, using ELISA (left) and iPCR (right). For each tooth, the mean value of 2 independent experiments was represented. Dotted line are the cut-off for ELISA (OD = 0.196.) and for iPCR (ΔCt = 16.13). Median, 25% and 75% percentiles are represented for each group and values are the following: by ELISA, negative teeth (0.143, 0.102, and 0.159), historically positive teeth (0.146, 0.130, and 0.174), historically positive teeth and positive by PCR (0.145, 0.115, and 0.158). By iPCR: negative teeth (13.01, 12, and 14.66), historically positive teeth (15.59, 14.29, and 19.53) and historically positive teeth and positive by PCR (0.145, 0.115, and 0.158). B: Comparison of iPCR, ELISA and PCR sensitivities for the detection of the *Y. pestis* antigen in ancient teeth.

Blind screening of ancient teeth for the detection of the *Y. pestis* antigen by ELISA and iPCR: Forty-six dental pulp samples were coded and tested blindly using ELISA and iPCR in 2 rounds of experiments (Figure 3). Among the coded teeth, 34 were historically *Y. pestis*-positive teeth, 2 were blank controls and 10 were *Y. pestis*-negative teeth, including 5 modern and 5 ancient teeth.

Among the 46 teeth tested using ELISA, 4 teeth exhibited an OD value above the cut-off value of 0.196 (Figure 3A). Among those four positive teeth, 3 teeth belonged to the 34 historically positive teeth, resulting in a sensitivity of 9% (3/34) for the detection of the *Y. pestis* antigen in the dental pulp (Figure 3A). The fourth tooth belonged to the 10 negative teeth group, leading to a specificity of 90%. Among the 3 positive teeth detected, 2 were extracted from skeletons excavated from the Lariey site (17^th^ century) and one from the Sens site (5^th^–6^th^ centuries). The 2 blank controls were negative, with OD values below the cut-off value (0.093±0.007 and 0.131±0.019, respectively). By ELISA, the comparison of negative teeth and historically positive teeth did not reveal a significant difference (p = 0.327).

**Figure 4:**
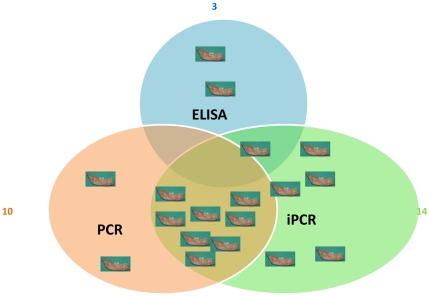
Venn diagram representing the number of positive teeth detected by PCR, iPCR and ELISA.

Among the 46 dental pulp samples tested using iPCR, 14 teeth exhibited a ΔCt above the cut-off value of 16.13 (Figure 3A). All of the 14 positive teeth belonged to the historically positive *Y. pestis* teeth group, resulting in a sensitivity of 41% (14/34), which was significantly higher than the sensitivity of the ELISA (p = 0.0021) (Figure 4 B). Blank controls exhibited ΔCt values below the cut-off value, and all of the negative teeth yielded a negative result, for a specificity of 100%. The 14 positive teeth detected are significantly positive compared to the negative teeth group (p = 0.0018). The 14 positive teeth by iPCR were from several sites: 5 teeth (36%) from the Lariey site (17^th^ century), 4 teeth (29%) from the Bourges site, 2 teeth (14%) from the Sens site (5^th^–6^th^ century), 2 teeth (14%) from the Bondy site (11^th^–15^th^ centuries) site and, finally, one tooth (7%) from the Venice site (14^th^ century). By PCR, *Y. pestis* DNA was detected in 10/34 teeth, leading to a sensitivity of 29%, which was significantly higher than the ELISA sensitivity (p = 0.03) but lower than the iPCR sensitivity (p = 0.31) (Figure 3B). In addition, *Y. pestis* DNA was not found by PCR in any of the 3 positive teeth that were detected using ELISA (Figure 4). However, PCR detected *Y. pestis* DNA in 57% (8/14) of positive teeth that were detected using iPCR. Among the 14 positive teeth detected using iPCR, only one tooth also tested positive using ELISA and iPCR, and no teeth tested positive using ELISA, iPCR and PCR (Figure 4).

The combination of iPCR and PCR led to the detection of 16/34 positive teeth, yielding a sensitivity of 47%, which was not significantly better than iPCR (p = 0.625) or PCR (p = 0.134) alone. Eight teeth detected positive using both PCR and iPCR, whereas 2 teeth and 6 teeth were only detected using PCR or iPCR, respectively (Figure 4). However, the combination of iPCR, PCR and ELISA allowed the detection of 18/34 positive teeth. Surprisingly, 2 teeth were only detected using ELISA (Figure 4).

## Discussion

Here, we describe for the first time the adaptation of iPCR for the detection of the *Y. pestis* antigen in dental pulp extracted from ancient teeth. The detection limit that was obtained by iPCR when testing the *Y. pestis* antigen in PBS indicated an improvement by a factor of 70 over the detection limit of the classical ELISA. One of the major drawbacks of iPCR is the presence of high background and non-specific binding. However, in our study, a specificity of 100% was determined using a predetermined cut-off. Among the 34 historically positive teeth that were collected from 5 different archeological sites, 41% were detected positive by iPCR, compared to 29% by PCR with *Y. pestis* confirmed in 14 teeth by previous detection of DNA by PCR and antigenic proteins detection by iPCR.

**Figure 5:**
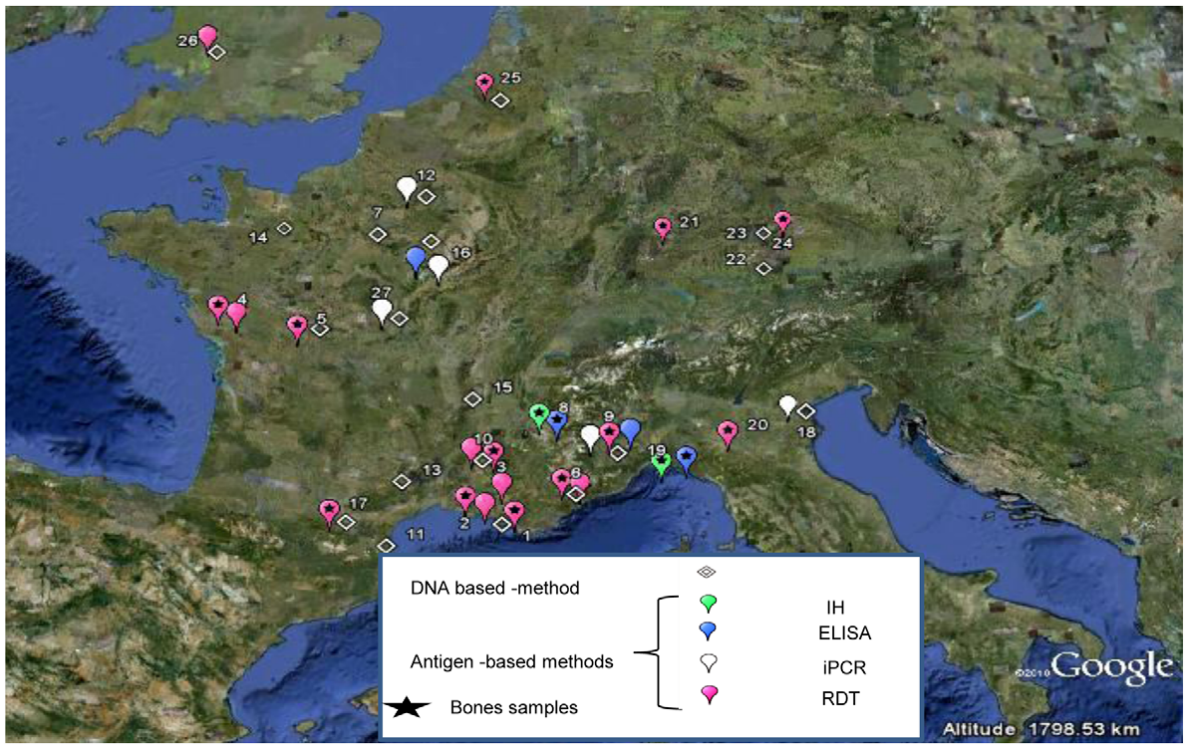
Molecular (losenge) and immunological (bubbles) detection of the plague agent *Yersinia pestis* in ancient burial sites in Europe in bones (star) and dental pulp (without star). For immunological detection, pink bubbles represent Rapid Diagnostic Test (RDT) detection, white bubbles represent iPCR detection, green bubbles represent immunohistochemical (IH) detection and purple bubbles represent ELISA detection. 27 burial sites are represented and references are between brackets: 1. Marseille [15], [23], [31], [41]; 2. Martigues [23], [41]; 3. Etang de Berre [31]; 4. La Chaize- le-Vicomte [42]; 5.Poitiers ([42]; 6. Draguignan [23], [31]; 7. Saint-Maurice [43]; 8.Briançon [24]; 9.Lariey ([31], present study); 10. Lambesc [15], [23], [31]; 11. Vilarnau [44]; 12. Bondy ([32], present study); 13. Montpellier [16], [33]; 14. Dreux [33]); 15.Vienne [41]; 16. Sens ([33], present study); 17.Saint-Laurent [19]; 18. Venise ([19], present study); 19.Genoa [24]; 20.Parme [19]; 21.Stuttgart [34]; 22. Aschheim [17]; 23.Manching-Pichl [45]; 24.Augsburg [19]; 25. Bergen-op-Zoom [19]; 26. Hereford [19]; 27.Bourges (present study).

The new methods herein developed, confirmed the presence of *Y. pestis* Black Death in human remains at 5 different archeological sites scattered over two countries with a broad time span from the 5^th^ to 17^th^ centuries [18], [29]–[33] (Figure 5).

Moreover, this is the first report of the presence of *Y. pestis* for the Bourges site by the detection of *Y. pestis* antigen.

Protein-based methods are considered more suitable for detecting the plague in historical samples because proteins are more resistant to environmental degradation than DNA [34]. The impact of the environment on DNA conservation has been demonstrated by Hoss *et al.*, who demonstrated inverse correlations between both the average temperature of the archeological site and the humidity levels and DNA retrieval [35]. In addition, the impact of tooth storage in soil has been investigated, demonstrating a decrease in extractable DNA by 90% after only 6 weeks of storage [36].

In summary, we successfully adapted for the first time an iPCR method to detect pathogen-derived antigens in ancient samples. Our results suggest that DNA and antigen-based methods are complementary. The double identification of the causative agent of the Black Death using antigen and DNA detection allows the resolution of controversies concerning the plague agent [37], [38]. Including this study, samples from 27 sites across 5 countries in Europe have been found positive by DNA or/and antigen detection of *Y. pestis* (Figure 5).

## Materials and Methods

### Case definition

**Figure 6:**
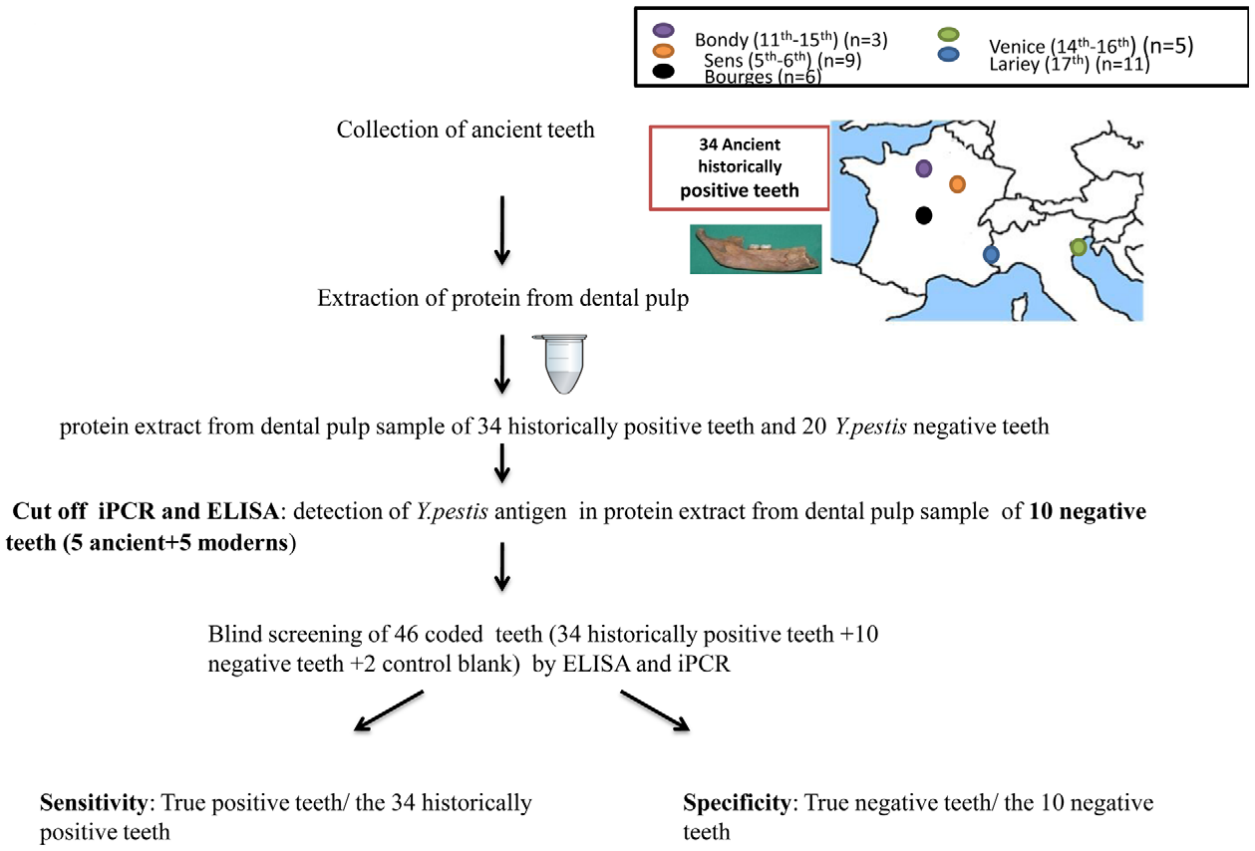
Strategy to detect the *Y. pestis* antigen in ancient specimens by ELISA and iPCR. In total, 34 ancient teeth were collected from skeletons that were excavated at five archaeological sites covering a broad time span from the 5^th^ to 16^th^ centuries. Dental pulp was extracted from 34 historically positive teeth and 20 negative teeth, which included modern and ancient teeth. The cut-off values for ELISA and iPCR were determined based on the screening of 10 negative teeth. Finally blind screening of 46 coded teeth, including the 34 historically positive teeth, 10 negative teeth and 2 blank controls was performed.

As negative controls, twenty negative teeth (10 modern and 10 ancient) were included in this study. Modern negative teeth were defined as teeth collected from healthy patients seeking dental care at a private clinic in Lille (France) and at the dental center Gaston Berger in Marseille (France). Ancient negative teeth were defined as teeth collected from skeletons of individuals without anthropologic and macroscopic evidence of *Y. pestis* infection that were excavated from a cemetery in Moirans, France (16th–18th). We defined historically *Y. pestis*-positive teeth, these collected from skeletons excavated from burial sites containing victims of the plague epidemic at which *Y. pestis* DNA was previously found in at least one tooth in the grave [19], [31], [32], [34]. Thirty-four historically positive teeth were collected from 5 archaeological sites with a broad time span from the 5^th^ to 16^th^ centuries (Figure 6) for this study. These teeth were analyzed using multiple molecular detection techniques as previously described [39]. All of the negative controls tested negative. Two blank controls contained PBS in place of dental pulp and were submitted to the same protocol as the teeth.

### Strategy

In the first set of experiments was compared to ELISA testing (Figure 1) for *Y. pestis* antigens serially diluted in PBS to concentrations of 7 µg to 70 pg and directly immobilized in the wells of a microplate. Then different concentrations of coated dental pulp were tested, and the concentration of the detection antibody was optimized, and ten negative teeth (5 modern and 5 ancient teeth) were used to determine the cut-off value (Figure 6).

Then 46 teeth (34 historically positive; 10 negative) were coded and tested blindly by ELISA and iPCR in 2 rounds of experiments as well as 2 were blank controls. Protein extraction from the dental pulp: Proteins were extracted by incubating the collected dental pulp as previously described [11] using 1 mL of 500 mM EDTA, pH 8.0, with agitation at room temperature for 24 hours. The suspension was then sonicated five times (1 min each) and centrifuged at 17,900× g for 40 min at room temperature. The suspension was dialyzed overnight in 2 L of a solution containing 50 mM Tris-HCl, pH 8.0, and 150 mM NaCl. The protein concentration was determined using the Bradford protein quantification protocol (Bradford, 1976).

### ELISA

TopYield microtiter modules (Chimera Biotec GmbH, Dortmund, Germany) were coated with 0.4 µg/well of each protein extract in 100 µl of carbonate buffer (0.05 M carbonate buffer, pH 9.6) overnight at 4°C. Unbound antigens were removed by washing with PBS containing 0.01% Tween 20 (PBST) three times. The plate was blocked for 1 hour with 5% milk powder in PBST. After washing with PBST three times, a mouse polyclonal antibody against *Y. pestis* that was produced in our laboratory was added (1∶400) in PBST containing 3% milk and incubated with gentle shaking for 1 hour. The specificity of the polyclonal antibody for *Yersinia* was assessed by immunofluorescence. The plate was washed with PBST 3 times and incubated with an anti mouse-IgG coupled to peroxidase (1∶1000) in PBST containing 3% milk with gentle shaking for 1 hour. After three washes with PBST, 200 µl of o-phenylenediamine dihydrochloride was added to each well for 10 min. The absorbance value at 485 nm was measured using a microtiter plate reader.

### Immuno-PCR

As for ELISA, 0.4 µg of the protein extract in 30 µl of coating buffer (Chimera Biotec GmbH, Dortmund, Germany) was used to coat Nunc TopYield microtiter modules (Chimera Biotec GmbH, Dortmund, Germany) overnight. Washing buffers A and B, coating buffer, blocking solution, conjugate dilution buffer (CDB), biotin-free CDB (CDB-b), anti–biotin-DNA conjugate antibody (CHI-biotin) and the master mix were provided in the Imperacer CHI biotin Kit (Chimera Biotec). Unbound dental pulp proteins were removed by washing three times with buffer A, and the modules were blocked by incubation with 240 µl of blocking solution for 30 minutes. The plate was then washed three times with buffer B at room temperature with orbital shaking.

For the detection of *Y. pestis* protein, 30 µl of mouse polyclonal antibody anti-*Y. pestis* previously diluted to 1∶400 was incubated in each well for 1 hour under orbital shaking. Unbound components were removed by three washes in buffer B. Biotinylated goat anti-mouse IgG, which was diluted 1∶1,000 in CDB, was added for 1 hour. To eliminate the unbound biotinylated detection antibody, three washes in buffer B were performed. The anti-biotin antibody-DNA conjugate was diluted (1∶600) in CDB-b and incubated for 45 minutes with gentle orbital shaking. To eliminate contamination and unbound components, three 30-sec washes and three 4-min washes with buffer B were followed by two 1-min washes with buffer A.

For quantitative PCR, 30 µl of the master mix was added to each well, and the modules were sealed using an optical adhesive. PCR was performed in an ABI PRISM 7900 HT with FAM as a fluorophore according to the following program provided by Chimera Biotec: 5 min at 95°C followed by 40 cycles of denaturation at 95°C for 12 s, annealing at 50°C for 30 s, and elongation at 72°C for 30 s. The background fluorescence threshold that differentiated negative from positive reactions was set immediately above the threshold for negative controls.

### Data analysis

For each group of teeth (negative and historically positive), a mean value with a standard error was calculated. Based on the negative teeth, a cut-off value was calculated as the mean value of the negative teeth+2SD for ELISA and the mean value minus 2SD for iPCR. For iPCR, the Ct values were inversely proportional to the amount of DNA template and, therefore, to the antigen concentration. Therefore, the Ct values were subtracted from the maximum number of cycles in the PCR, which was typically 40 cycles, to generate ΔCt values, which were used to compare iPCR to ELISA. The sensitivity and specificity of ELISA and iPCR were calculated as previously described [40]. For the statistical analysis, the Chi^2^ test with GraphPad InStat version 3.0b software was used to compare the sensitivities of iPCR, ELISA and PCR. A Man Whitney non parametric test was performed to compare the positive and the negative teeth groups.

### Ethic statement

As negative controls, twenty negative teeth (10 modern and 10 ancient) were included in this study. Modern human teeth were provided by Dr Pierre ELIA (Lille, France) and by Thi-Nguyen-Ny Tran (Marseille, France) after obtaining informed written consent from the patients. A copy of the written consent is available for review by the Editor-in-Chief of this journal. This study was approved by the Ethic Committee, Institute Fédératif de Recherche 48, Marseilles, France.

Ancient human teeth were provided by the following anthropologists, all co-authors of the manuscript: Dominique Castex (Laboratoire d'Anthropologie, Bordeaux, France), Cyrille Le Forestier (Institut National de Recherches Archéologiques Préventives, France), Michel Signoli (Laboratoire d'Anthropologie, Marseille, France). According to the French law, no permission is required for scientific investigations of old mass graves. Under French law (http://legifrance.gouv.fr/affichTexte.do?cidTexte=JORFTEXT000000221337, Loi n° 2001-44 du 17 janvier 2001 relative à l'archéologie préventive.), the remains (even humans) from these periods are considered as archaeological remains like for the other remains (pottery, coins …). The anthropologists, the archaeologists because of their position, are free to take samples and to analyse them for their scientific research and free to analyse these samples by any technique they need to use, for scientific purposes. Each one of the three anthropologists was responsible for the work carried out at the archeological sites that he studied.
